# Mercurial-resistance determinants in *Pseudomonas* strain K-62 plasmid pMR68

**DOI:** 10.1186/2191-0855-3-41

**Published:** 2013-07-28

**Authors:** Yuka Sone, Yusuke Mochizuki, Keita Koizawa, Ryosuke Nakamura, Hidemitsu Pan-Hou, Tomoo Itoh, Masako Kiyono

**Affiliations:** 1 Department of Public Health and Molecular Toxicology, School of Pharmacy, Kitasato University, 5-9-1 Shirokane, Tokyo, Minato-ku 108-8641, Japan; 2 Faculty of Pharmaceutical Sciences, Setsunan University, 45-1 Nagaotoge-cho, Osaka, Hirakata 573-0101, Japan

**Keywords:** Mercury resistance, *mer* operon, Plasmid pMR68, *Pseudomonas* strain K-62

## Abstract

We report the complete nucleotide sequence of plasmid pMR68, isolated from *Pseudomonas* strain K-62, two plasmids contribute to broad-spectrum mercury resistance and that the *mer* operon from one of them (pMR26) has been previously characterized. The plasmid was 71,020 bp in length and contained 75 coding regions. Three *mer* gene clusters were identified. The first comprised *merR-orf4-orf5-merT1-merP1-merF-merA-merB1*, which confers bacterial resistance to mercuric ions and organomercury. The second and third clusters comprised *merT2-merP2*, which encodes a mercury transport system, and *merB2*, which encodes an organomercurial lyase, respectively. The deduced amino acid sequences for the proteins encoded by each of the *mer* genes identified in pMR68 bore greater similarity to sequences from *Methylobacterium extorquens* AM1 than to those from pMR26, a second mercury-resistance plasmid from *Pseudomonas* strain K-62. *Escherichia coli* cells carrying pMKY12 (containing *merR-orf4-orf5-merT1-merP1-merF-merA-merB1* cloned from pMR68) and cells carrying pMRA114 (containing *merR-merT-merP-merA-merG-merB1* cloned from plasmid pMR26) were more resistant to, and volatilized more, mercury from mercuric ions and phenylmercury than the control cells. The present results, together with our earlier findings, indicate that the high phenylmercury resistance noted for *Pseudomonas* strain K-62 seems to be achieved by multiple genes, particularly by the multiple *merB* encoding organomercurial lyase and one *merG* encoding cellular permeability to phenylmercury. The novel *mer* gene identified in pMR68 may help us to design new strategies aimed at the bioremediation of mercurials.

## Introduction

*Pseudomonas* strain K-62, a bacterial strain isolated from phenylmercury-polluted soil, is about 1,000 times more resistant to phenylmercury than sensitive strains of *Escherichia coli* (Tonomura et al. 1968). A study performed about 40 years ago showed that the biochemical mechanism underlying this mercurial resistance is based on the enzymatic degradation of organomercurials and the subsequent reduction of the resulting mercuric ions to the less toxic and more volatile metallic mercury (Tonomura et al. 1968). Two separate organomercurial lyases, designated S-1 and S-2, each showing somewhat different physical properties and substrate specificities, are thought to be responsible for the resistance of *P.* K-62 to phenylmercury (Tezuka and Tonomura 1976;Tezuka and Tonomura 1978). The organomercurial resistance of this soil strain is encoded by two plasmids, pMR26 and pMR68 (Kiyono et al. 1995b). In addition, pMR26 contains two *mer* operons that map about 1 kb apart (Kiyono et al. 1997). One comprises *merR-*o/p-*merT*-*merP*-*merA*-*merG*-*merB1*. The other is a defective *mer* operon comprising *merR*-o/p-*merB2-merD* (Kiyono and Pan-Hou 1999a).

Studies suggest that *merR* is a regulatory gene that both negatively and positively controls the transcription of *merTPABD* (Ansari et al. 1995;Brown et al. 2003), whereas *MerD* is a transcriptional co-regulator (Hobman and Brown 1997;Kiyono et al. 1995a;Lund and Brown 1987). *MerT*, *merP*, *merA*, and *merB* encode a membrane Hg^2+^-transport protein (Hobman and Brown 1997;Kiyono et al. 1995a;Lund and Brown 1987), a periplasmic Hg^2+^-binding protein (Hobman and Brown 1997;Kiyono et al. 1995a;Kiyono et al. 2000;Lund and Brown 1987), a mercuric reductase (Hobman and Brown 1997; Schiering et al. 1991; Silver and Phung 1996), and a organomercurial lyase (Griffin et al. 1987;Lafrance-Vanasse et al. 2009;Miller 1999), respectively. *merG*, identified in pMR26, is a newly-identified *mer*-gene involved in phenylmercury resistance, which is thought to act by reducing cell permeability to phenylmercury (Kiyono and Pan-Hou 1999b). Taken together, these findings suggest that the high resistance to phenylmercury shown by *P.* K-62 is mediated by the two functional organomercurial lyase enzymes encoded by pMR26 *merB1* and *merB2* (Kiyono et al. 1995b; Kiyono and Pan-Hou 1999a), by changes in cellular permeability to phenylmercury mediated by *merG* (Kiyono and Pan-Hou 1999b), and by an presumptive *mer* operon located on plasmid pMR68 (Kiyono et al. 1995b) However, no mercurial-resistance loci have been identified in pMR68; indeed, a previous study shows that removal of pMR26 from strain K-62 does not alter its mercurial-resistant phenotype nor prevent it from volatilizing Hg^2+^ and organomercurials (Kiyono et al. 1995b).

To fully explain the high resistance to phenylmercury observed in this strain of soil bacteria, it is essential to understand the *mer* genes expressed by pMR68. The aim of the present study was to completely identify the mercury resistance genes of plasmid pMR68 isolated from strain K-62. The *mer* genes encoded by pMR68 were then cloned and analyzed.

## Materials and methods

### Bacterial strains and culture conditions

*P.* strain K-62, isolated from phenylmercury-polluted soil in Japan (Tonomura et al. 1968) and deposited in a culture collection belonging to the WDCM56, was kindly supplied by Dr. K. Tonomura and grown in nutrient broth as previously described (Tezuka and Tonomura 1976) (see Table 1). *E. coli* XL1-Blue was grown at 37°C in Luria-Bertani (LB) medium. Antibiotics or mercuric chloride were added to the medium at the following concentrations when appropriate: ampicillin, 100 μg/ml (*E. coli*); mercuric chloride, 40 μg/ml.

**Table 1:** Strains and plasmids used in this study

**Stains and plasmids**	**Description or relevant feature(s)**		**Reference or source**
Strains			
*E. coli* XL1-Blue	*recA1 endA1 gyrA96 thi hsdR17 supE44 relA1 lac/* [F’::Tn *10 proAB+ lacI*q *lacZM15 traD36*]		(Bullock et al. 1987)
*P.* strain K-62 (wild)	82, 68,56, 31, 26, 8.5 kb plasmids	IC50 of mercuric chloride; 100 ppm, mercury vapor activity; +	Kiyono et al. (1995b)
*P.* strain K-62 (mutant 26)	82, 68, 56, 31, 8.5 kb plasmids	IC50 of mercuric chloride; 50 ppm, mercury vapor activity; +	Kiyono et al. (1995b)
*P.* strain K-62 (mutant 68)	82, 56, 31, 26, 8.5 kb plasmids	IC50 of mercuric chloride; 17 ppm, mercury vapor activity; +	Kiyono et al. (1995b)
*P.* strain K-62 (mutant TY)	82, 56, 31, 8.5 kb plasmids	IC50 of mercuric chloride; 2 ppm, mercury vapor activity; -	Kiyono et al. (1995b)
Plasmids			
pMR26	26 kb plasmids from *P.* strain K-62		(Kiyono et al. 1997)
pMR68	68 kb plasmids from *P.* strain K-62		This study
pUC118	None; cloning vector		(Vieira et al. 1987)
pMRA17	*merR-o/p-merT-merP-merA-merG-merB1* of pMR26 in pBluescriptII		(Kiyono et al. 1997)
pMRA114	*merR-o/p-merT-merP-merA-merG-merB1* of pMR26 in pUC118		This study
pMKY12	*merR-o/p-orf4-orf5-merT1-merP1-merA-merB1* of pMR68 in pUC118		This study

### DNA purification

Six plasmids (8.5–82 kb) (Kiyono et al. 1995b) were purified from strain K-62 grown in nutrient broth for 3 days at 30°C according to the method of Sasakawa et al. (Sasakawa et al. 1986). The purified plasmids were loaded into the wells of a 0.7% low-melting temperature agarose gel (SeaPlaque GTG agarose, Lonza Rockland, Inc., Rockland, ME) and electrophoresed in 0.5 × TBE (45 mM Tris HCl, 45 mM boric acid, 1 mM EDTA) in a contour clamped homogeneous electric field (CHEF) DRII device (Bio-Rad Laboratories, Hercules, CA) with the pulse-time ramped from 10 to 60 s (6 V/cm) for 16 h at 14°C. The gels were then stained with ethidium bromide. The band corresponding to the pMR68 plasmid was excised and equilibrated in small amount of buffer (10 mM Tris HCl pH 7.5, 0.25 mM EDTA, 100 mM NaCl). The agarose was melted at 68°C and digested with ß-agarase (New England Biolabs, Hertfordshire, England). The pMR68 plasmid was then concentrated by ethanol precipitation.

### DNA sequencing

Purified pMR68 plasmid DNA (15 μg) was sequenced by Eurofins MWG Operon (Ebersberg, Germany) using a Genome Sequencer FLX Titanium system (Roche, Basel, Switzerland). Shotgun sequencing was then performed on the range of one region of a 16-region picotiter plate, resulting in 33,964 reads with an average length of 340 bp. The sequences were assembled using Celera Assembler Version 5.3, generating 18 contigs of at least 1,000 bp in length, some of which were high coverage (>100-fold). Connections between these contigs and adjacent contigs with similarly high coverage were identified by looking for sequencing reads that were split between contigs by the assembly software. Using this process, a chain of seven contigs was assembled into a 71,020 bp sequence. The joins between the seven contigs were checked by PCR using the following primer pairs: 1U-68 kb-15850 and 8 L-68 kb-16600; 2U-68 kb-18520 and 9 L-68 kb-19216; 3U-68 kb-19421 and 10 L-68 kb-20259; 4U-68 kb-38451 and 11 L-68 kb-39477; 5U-68 kb-42220 and 12 L-68 kb-43396; 6U-68 kb-67929 and 13 L-68 kb-68480; and 7U-68 kb-70484 and 14 L-68 kb-457 (see Additional file 1: Table S1).

After the complete nucleotide sequence of pMR68 was obtained, potential open reading frames (ORFs) was searched using the program of genetic information processing software (Genetyx corporation, Tokyo, Japan) and using protein BLAST (http://blast.ncbi.nlm.nih.gov/Blast.cgi) to confirm the results. Conserved domains were identified searching for Clusters of Orthologous Groups of proteins (COGs) in the NCBI data base. The molecular weights of the encoded proteins were determined by ProtParam (Swiss Institute of Bioinformatics; http://www.expasy.ch/tools/protparam.html). The annotated sequence of pMR68 was deposited in the NCBI database under Accession No. NC019309.

### Gene cloning and analysis of the *mer* operon

Plasmid pMKY12 was constructed as follows: Plasmid pMR68 (accession no. NC019309) was used as the template for PCR amplification (PrimeSTAR GXL DNA polymerase, Takara Bio, Inc., Otsu, Japan) of a 10.3 kb fragment containing the *merR-*o/p-*ofr4-orf5-merT1-merP1-merF-merA-merB1* genes. The primers used were 16U-68 kb-2393 and 21 L-68 kb-9566 (Additional file 1: Table S1). After blunting and 5'-phosphorylation of the DNA fragment using a Mighty Cloning Kit (Blunt End) (Takara Bio, Inc., Otsu, Japan), the DNA fragment was cloned into the blunt-ended (*Hin*c II) vector, pUC118 (Vieira and Messing 1987).

Plasmid pMRA114 was constructed as follows: Plasmid pMRA17, containing a 6.6 kb *merR-*o/p*-merT-merP-merA-merG-merB1* fragment from pMR26 (accession no. D83080), which contains restriction sites for *Sac*I, was used as the starting material. After digestion with *Sac*I, the 6.6 kb fragment was cloned into the corresponding sites in pUC118. The integrity of all cloned fragments was confirmed by sequencing.

### Mercury susceptibility tests and volatilization activity tests

The resistance of *E. coli* XL1-Blue carrying pUC118 (control vector), pMRA114, or pMKY12 to HgCl_2_ or C_6_H_5_HgOCOCH_3_ was determined in liquid medium. *E. coli* cells carrying the control or recombinant plasmids were grown overnight in LB medium at 37°C. Cells were harvested and suspended in LB medium (1.6 × 10^7^ cells/200 μL/well) containing HgCl_2_ or C_6_H_5_HgOCOCH_3_ at various concentrations. After incubation at 37°C for 16 h, the absorbance of each culture was read at A_600_ to measure cell growth.

The mercury volatilization assay was performed as follows: *E. coli* cells carrying the control or recombinant plasmids were grown to mid-exponential phase and then suspended in LB medium containing 50 μM HgCl_2_ or 5 μM C_6_H_5_HgOCOCH_3_. After incubating at 37°C for 16 h, the samples were digested with concentrated nitric acid for 2 h at 90°C and the amount of mercury remaining in the medium was determined by flameless cold-vapour atomic adsorption spectrometry using an atomic mercury analyser (HG-310; Hiranuma, Japan).

## Results

### General features of plasmid pMR68

**Figure 1:**
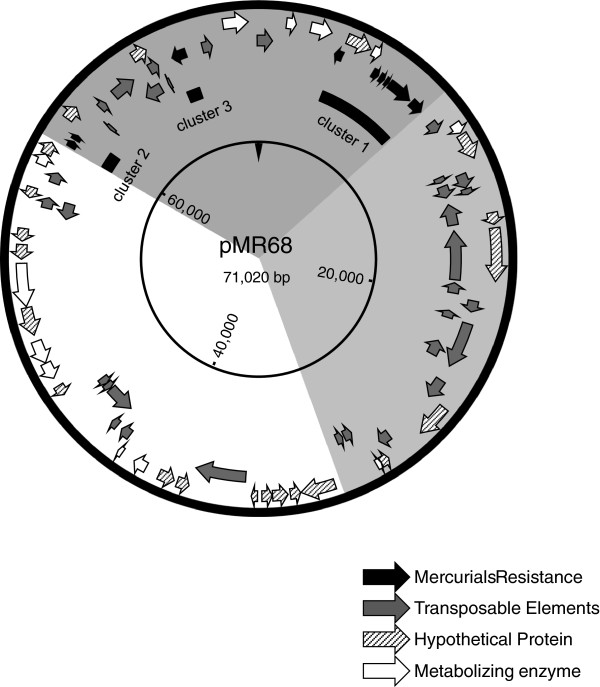
**Genetic map of the *P. *strain K-62 plasmid, pMR68.** The deduced coding regions are shown by open arrows indicating the direction of transcription. The circular positions are indicated at intervals of 20,000 bp. The gene clusters responsible for mercurial resistance, transposable elements, metabolizing enzymes, and hypothetical proteins are indicated by the black, gray, open and shaded arrows, respectively.

The complete nucleotide sequence of plasmid pMR68 was assembled into a circular DNA sequence comprising 71,020 bp, with an overall G + C content of 64.5%. The sequence showed that pMR68 was 3 kb larger than previously calculated on the basis of agarose gel electrophoresis (Kiyono et al. 1995b). Figure 1 shows a detailed genetic map of pMR68. The predicted coding regions showed a particular genetic organization, highlighting two well-defined regions that corresponded to genomic islands (comprising 41 kb of the complete plasmid). The first region possessed a 21 kb island (pMR68 co-coordinates: 59,285–71,020 and 1–9,472), which contained genes involved in mercurial resistance and mobility (Figure 1). The second region possessed a 20 kb island (pMR68 co-coordinates; 10,189–30,567), which contained plasmid transposable elements (Figure 1). A summary of the sequence data for pMR68, including the length and molecular mass of the predicted proteins and their sequence homology with known proteins, is shown in Additional file 2: Table S2.

### Mercury-resistance (Hg^R^) determinants

**Figure 2:**
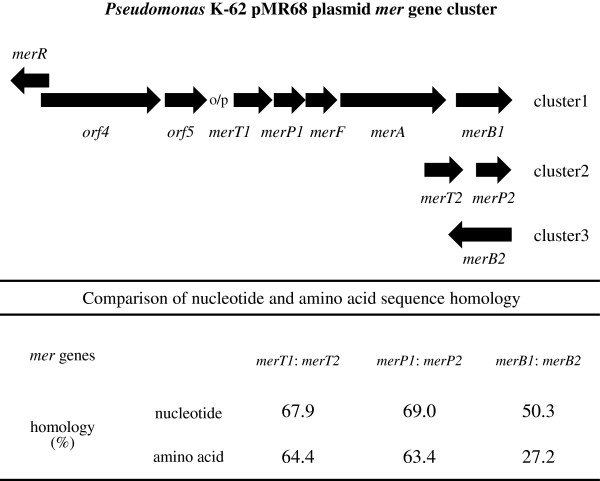
**Organization of the *mer *operon of pMR68 and its homology with the corresponding *mer *genes identified in pMR68.***merR*, regulatory gene; *merT and merF*, mercury transport genes; *merP*, mercury binding gene; *merA*, mercuric reductase gene; *merB*, organomercurial lyase gene; *orf*, unknown open reading frame.

The pMR68 plasmid contained three putative mercury-resistance (*mer*) gene clusters (Figure 1): Cluster 1) a potential *mer* operon, consisting of the *merR-orf4-orf5-T1-P1-F-A-B1* genes (ORFs 3 and 6–10); Cluster 2) mercury transport genes, *merT2-P2* (ORFs 62 and 63); and Cluster 3) an organomercurial lyase gene, *merB2* (ORF 72) (Additional file 2: Table S2 and Figures 1 and 2). The potential *mer* operon within pMR68 was located between putative transposable elements, and flanked by genes encoding a transposase IS4 family protein (ORF 75) and a transposase IS5 family protein (ORF 11) (Additional file 2: Table S2). The incomplete *mer* operon (cluster 2) within pMR68 was also located between putative transposable elements, and flanked by genes encoding a transposase IS30 family protein (ORF 56) and a transposase IS116 family protein (ORF 68) (Additional file 2: Table S2). The DNA sequence showed the presence of putative promoters upstream of the *merT1* genes within the complete *mer* operon of cluster 1 (Figure 2). Upstream of *merR* and upstream of *merT1* were sequences containing potential −35 (ATCAGA) and −10 (GATTAT) and −35 (TTGCAC) and −10 (CATAAT) sequences, and a dyad symmetrical sequence (GCACCTGTAGCCGCTACAGGTTG), respectively, which could be interpreted as an operator/promoter (o/p) sequence (cluster 1, Figure 2).

ORF4 (encoding a hypothetical protein (HP)) and ORF5 (showing 63% identity to phosphoribosyl-AMP cyclohydrolase from *Parvibaculum lavamentivorans* DS-1) were located between MerR and MerT1. Two sets of *merT*, *merP*, and *merB* genes were found in pMR68, and the similarities between *merT1* and *merT2*, *merP1* and *merP2*, and *merB1* and *merB2* ranged from 27% to 69% at both the nucleic acid and amino acid levels (Figure 2). The sequence similarity between the pMR68 and pMR26 proteins (Kiyono et al. 1997;Kiyono and Pan-Hou 1999a) from strain K-62 or the well-known pDU1358 (Griffin et al. 1987) and Tn*21* (Gilbert and Summers 1988;Liebert et al. 1999) proteins from Gram-negative bacteria was low (Table 2)*.*

**Table 2:** **Amino acid sequence homology of *mer *genes between pMR68 and pMR26, pDU1358, Tn*21 *or *M. extorquens *AM1 putative *mer *operon**

**% of amino acid sequence homology**									
**pMR68**	**MerR**	**MerT1**	**MerT2**	**MerP1**	**MerP2**	**MerF**	**MerA**	**MerB1**	**MerB2**
pMR26	32.4	38.8	47.0	31.3	36.0	-	35.4	21.3	22.0
pDU1358	32.5	38.8	45.5	31.2	36.0	-	26.7	21.0	19.7
Tn*21*	31.8	38.8	47.0	31.1	38.7	-	38.6	-	-
*M. extorquens* AM1	73.0	76.0	71.0	76.0	67.0	-	84.0	-	-

### Gene cloning and analysis of the *mer* operon

**Figure 3:**
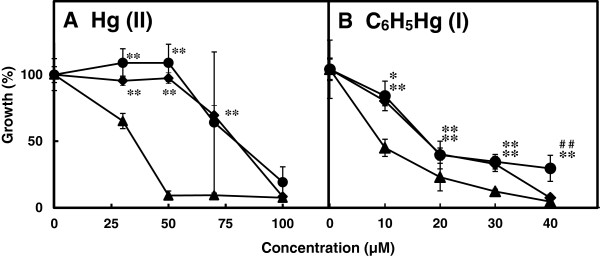
**Susceptibility to mercurials.***E. coli* cells carrying pUC118 (filled triangles), pMKY12 (filled circles), or pMRA114 (filled diamonds) were grown in liquid medium containing varying concentrations of HgCl_2_**(A)** or C_6_H_5_HgOCOCH_3_**(B)**. Growth was estimated by measuring the turbidity at 600 nm. Data represent the mean ± S.D. of triplicate measurements from three independent experiments. *p < 0.05 *vs.* control. **p < 0.01 *vs.* control. ^##^*p <* 0.01 *vs.* pMRA114.

To identify the physiological role played by the *mer* operons in pMR68 and pMR26 in *E. coli*, we constructed recombinant plasmids pMKY12 and pMRA114, which contained the *merR-orf4-orf5-merT1-merP1-merF-merA-merB1* genes from pMR68 and the *merR-merT-merP-merA-merG-merB1* genes from pMR26, respectively (Table 1). Bacteria containing pMKY12 showed greater resistance to Hg(II) than control cells carrying plasmid pUC118; the level of resistance was almost the same as that shown by cells containing pMRA114 (Figure 3A).

**Figure 4:**
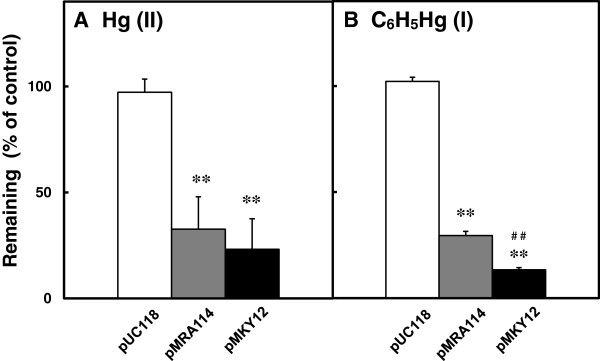
**Volatilization of mercury from Hg(II) and C**_**6**_**H**_**5**_**Hg(I).***E. coli* cells carrying pUC118 (empty bar), pMRA114 (shaded bar), or pMKY12 (black bar) were grown in liquid medium containing 50 μM HgCl_2_**(A)** or 5 μM C_6_H_5_HgOCOCH_3_**(B)**. After incubation at 37°C for 16 h, the amount of mercury remaining in the medium was measured as described in “Materials and Methods”. Data represent the mean ± S.D. of triplicate measurements from three independent experiments. **p < 0.01 *vs.* control. ^##^*p <* 0.01 *vs.* pMRA114.

We next examined the volatilization of mercury from Hg(II) and C_6_H_5_Hg(I) by cells containing a control vector (pUC118) or the pMKY12 or pMRA114 plasmids. As shown in Figure 4, cells carrying both pMKY12 and pMRA114 were able to volatilize mercury from both Hg(II) and C_6_H_5_Hg(I). Volatilization of Hg(II) was similar between cells carrying pMKY12 or pMRA114; however, volatilization of C_6_H_5_Hg(I) was significantly higher in cells carrying pMKY12 than in those carrying pMRA114 (Figure 4B). A volatilization test indicated that the cells carrying pMKY12 were able to volatilize almost 90% of the total phenylmercury.

## Discussion

The present study determined the complete nucleotide sequence of plasmid pMR68 (isolated from *P.* strain K-62). In addition, the *mer* genes in pMR68 and pMR26 were identified, sequenced, and cloned in *E. coli*. The pMR68 sequence contained 75 complete coding regions; however, we were not able to identify a predicted origin of replication (Additional file 2: Table S2 and Figure 1). Although most of the identified genes (44%) encoded mobile elements related to transfer functions, 12% encoded mercurial-resistance determinants, 16% encoded metabolism-related genes, and 28% encoded hypothetical proteins (HPs).

One of the three *mer-*gene clusters in pMR68 (*merR-orf4-orf5-merT1-merP1-merF-merA-merB1*) was identified as a potential *mer* operon, which confers bacterial resistance to both inorganic and organic mercury (Additional file 2: Table S2 and Figure 3). The number and order of the *mer* genes on this potential *mer* operon were different to those of *merR1-*o/p*-T-P-A-G-B1* of pMR26 (which encodes resistance to both inorganic and organic mercury) and *merR2-*o/p*-B2-D* of pMR26 (which confers bacterial hypersensitivity to organomercury compounds) (Table 2 and Figures 1 and 2) (Kiyono et al. 1997;Kiyono and Pan-Hou 1999a;Kiyono et al. 1997)*.* The deduced amino acid sequences for the proteins encoded by the *mer* genes in pMR68 were more similar (67–84%) to those of a putative *mer* operon in *Methylobacterium extorquens* AM1, which lacks the *merB* gene (Vuilleumier et al. 2009), than to those in pMR26 (21–47%) (Table 2).

We identified potential −35 and −10 sequences and a dyad symmetrical sequence lying upstream of pMR68 *merR* and upstream of *merT1,* respectively; these sequences may represent putative promoters of the *mer* operon of cluster 1 in pMR68. The distance between the −35 and −10 positions within the putative pMR68 *merR* promoter was 19 bp. The spacing between the *merR* promoter and Tn*21* in pMR26 was also 19 bp, which is essential for the “twist and bend” mechanism underlying transcriptional activation (Ansari et al. 1995;Kiyono et al. 1997;Kiyono and Pan-Hou 1999a). Taken together, these observations suggest that *merT1-P1-F-A-B1* is regulated by both the *merR* gene and the *mer* operator.

Volatilization of mercury from organomercurials is thought to result from the degradation of organic mercury by the organomercurial lyase encoded by *merB,* followed by the reduction of the resulting Hg^2+^ to volatile Hg^0^ by the mercuric reductase encoded by *merA* (Barkay et al. 2003;Silver and Phung le 2005). Cells carrying pMKY12 (containing *merR-orf4-orf5-merT1-merP1-merF-merA-merB1* cloned from pMR68) and cells carrying pMRA114 (containing *merR-merT-merP-merA-merG-merB1* cloned from plasmid pMR26) were more resistant to, and volatilized more, mercury from mercuric chloride and phenylmercuric acetate than the control cells (Figures 3 and 4).

The present results, together with those of our previous study, suggest that the high resistance to phenylmercury shown by strain K-62 may be due to the following: [1] the functional organomercurial lyase enzymes encoded by pMR26 *merB1* and *merB2* (Kiyono et al. 1995b;Kiyono et al. 1997;Kiyono and Pan-Hou 1999a), and pMR68 *merB1*; [2] the two functional mercuric reductases encoded by pMR26 *merA* (Kiyono et al. 1995b;Kiyono and Pan-Hou 1999a) and pMR68 *merA*; [3] the multi-functional transporters encoded by pMR26 *merT* and *merP* (Kiyono et al. 1995a;Kiyono et al. 2000;Nagata et al. 2006;Uno et al. 1997), and by pMR68 *merT1*-*merP1*-*merF*; and [4] alterations in cellular permeability to phenylmercury mediated by pMR26 *merG* (Kiyono and Pan-Hou 1999b)*.* Further analysis of *orf4* and *orf5* within pMR68 is currently on-going.

In conclusion, sequence analysis of pMR68 showed that the plasmid contains novel genes that may provide *Pseudomonas* strains with the means to adapt to a wide variety of challenging environments, including exposure to heavy metals. Such resistance mechanisms are likely to be linked to the evolution of the bacterial hosts. The novel *mer* gene identified in pMR68 may help us to design new strategies aimed at the bioremediation of mercury-containing compounds present in the environment.

## Competing interests

The authors declare that they have no competing interests.

## Supplementary Material

Additional file 1: Table S1 Oligonucleotide primers used in this study. Click here for file

Additional file 2: Table S2 Summary of location of predicted coding regions on plasmid pMR68. Click here for file

## References

[B1] Ansari AZ Bradner JE O'Halloran TV DNA-bend modulation in a repressor-to-activator switching mechanism Nature 1995 3 6520 371 375 10.1038/374370a0 7885478

[B2] Barkay T Miller SM Summers AO Bacterial mercury resistance from atoms to ecosystems FEMS Microbiol Rev 2003 3 2–3 355 384 10.1016/s0168-6445(03)00046-9 12829275

[B3] Brown NL Stoyanov JV Kidd SP Hobman JL The MerR family of transcriptional regulators FEMS Microbiol Rev 2003 3 2–3 145 163 12829265 10.1016/S0168-6445(03)00051-2

[B4] Bullock WO Fernandez JM Short JM XL1-Blue: A high efficiency plasmid transforming *recA Escherichia coli* strain with beta-galactosidase selection Biotechniques 1987 3 4 376 379

[B5] Gilbert MP Summers AO The distribution and divergence of DNA sequences related to the Tn*21* and Tn*501 mer* operons Plasmid 1988 3 2 127 136 10.1016/0147-619X(88)90015-7 2853392

[B6] Griffin HG Foster TJ Silver S Misra TK Cloning and DNA sequence of the mercuric- and organomercurial-resistance determinants of plasmid pDU1358 Proc Natl Acad Sci USA 1987 3 10 3112 3116 10.1073/pnas.84.10.3112 3033633 PMC304818

[B7] Hobman JL Brown NL bacterial mercury-resistance genes Met Ions Biol Syst 1997 3 527 568 9046583

[B8] Kiyono M Pan-Hou H DNA sequence and expression of a defective *mer* operon from *Pseudomonas* K-62 plasmid pMR26 Biol Pharm Bull 1999 3 9 910 914 10.1248/bpb.22.910 10513611

[B9] Kiyono M Pan-Hou H The *merG* gene product is involved in phenylmercury resistance in *Pseudomonas* strain K-62 J Bacteriol 1999 3 3 726 730 9922233 10.1128/jb.181.3.726-730.1999 PMC93436

[B10] Kiyono M Omura T Fujimori H Pan-Hou H Lack of involvement of *merT* and *merP* in methylmercury transport in mercury resistant *Pseudomonas* K-62 FEMS Microbiol Lett 1995 3 3 301 306 10.1111/j.1574-6968.1995.tb07540.x 7781979

[B11] Kiyono M Omura T Fujimori H Pan-Hou H Organomercurial resistance determinants in *Pseudomonas* K-62 are present on two plasmids Arch Microbiol 1995 3 4 242 247 10.1007/BF00393375 7763132

[B12] Kiyono M Omura T Inuzuka M Fujimori H Pan-Hou H Nucleotide sequence and expression of the organomercurial-resistance determinants from a *Pseudomonas* K-62 plasmid pMR26 Gene 1997 3 2 151 157 10.1016/S0378-1119(96)00741-X 9168120

[B13] Kiyono M Uno Y Omura T Pan-Hou H Role of MerT and MerP from *Pseudomonas* K-62 plasmid pMR26 in the transport of phenylmercury Biol Pharm Bull 2000 3 3 279 282 10.1248/bpb.23.279 10726879

[B14] Lafrance-Vanasse J Lefebvre M Di Lello P Sygusch J Omichinski JG Crystal structures of the organomercurial lyase MerB in its free and mercury-bound forms: insights into the mechanism of methylmercury degradation J Biol Chem 2009 3 2 938 944 10.1074/jbc.M807143200 19004822

[B15] Liebert CA Hall RM Summers AO Transposon Tn*21*, flagship of the floating genome Microbiol Mol Biol Rev 1999 3 3 507 522 10477306 10.1128/mmbr.63.3.507-522.1999 PMC103744

[B16] Lund PA Brown NL Role of the *merT* and *merP* gene products of transposon Tn*501* in the induction and expression of resistance to mercuric ions Gene 1987 3 2–3 207 214 3038684 10.1016/0378-1119(87)90047-3

[B17] Miller SM Bacterial detoxification of Hg(II) and organomercurials Essays Biochem 1999 3 17 30 10730186 10.1042/bse0340017

[B18] Nagata T Kiyono M Pan-Hou H Involvement of aromatic amino acids in phenylmercury trasport by MerT protein J Health Sci 2006 3 4 475 477 10.1248/jhs.52.475

[B19] Sasakawa C Kamata K Sakai T Murayama SY Makino S Yoshikawa M Molecular alteration of the 140-megadalton plasmid associated with loss of virulence and Congo red binding activity in *Shigella flexneri* Infect Immun 1986 3 2 470 475 3002985 10.1128/iai.51.2.470-475.1986 PMC262355

[B20] Schiering N Kabsch W Moore MJ Distefano MD Walsh CT Pai EF Structure of the detoxification catalyst mercuric ion reductase from *Bacillus* sp. strain RC607 Nature 1991 3 6331 168 172 10.1038/352168a0 2067577

[B21] Silver S le Phung T A bacterial view of the periodic table: genes and proteins for toxic inorganic ions J Ind Microbiol Biotechnol 2005 3 11–12 587 605 10.1007/s10295-005-0019-6 16133099

[B22] Silver S Phung LT Bacterial heavy metal resistance: new surprises Annu Rev Microbiol 1996 3 753 789 10.1146/annurev.micro.50.1.753 8905098

[B23] Tezuka T Tonomura K Purification and properties of an enzyme catalyzing the splitting of carbon-mercury linkages from mercury-resistant *Pseudomonas* K-62 strain. I. Splitting enzyme 1 J Biochem 1976 3 1 79 87 9382 10.1093/oxfordjournals.jbchem.a131261

[B24] Tezuka T Tonomura K Purification and properties of a second enzyme catalyzing the splitting of carbon-mercury linkages from mercury-resistant *Pseudomonas* K-62 J Bacteriol 1978 3 1 138 143 27499 10.1128/jb.135.1.138-143.1978 PMC224790

[B25] Tonomura K Maeda K Futai F Nakagami T Yamada M Stimulative vaporization of phenylmercuric acetate by mercury-resistant bacteria Nature 1968 3 5129 644 646 4866536 10.1038/217644b0

[B26] Uno Y Kiyono M Tezuka T Pan-Hou H Phenylmercury transport mediated by *merT-merP* genes of *Pseudomonas* K-62 plasmid pMR26 Biol Pharm Bull 1997 3 1 107 109 10.1248/bpb.20.107 9013821

[B27] Vieira J Messing J Production of single-stranded plasmid DNA Methods Enzymol 1987 3 3 11 3323803 10.1016/0076-6879(87)53044-0

[B28] Vuilleumier S Chistoserdova L Lee MC Bringel F Lajus A Zhou Y Gourion B Barbe V Chang J Cruveiller S Dossat C Gillett W Gruffaz C Haugen E Hourcade E Levy R Mangenot S Muller E Nadalig T Pagni M Penny C Peyraud R Robinson DG Roche D Rouy Z Saenampechek C Salvignol G Vallenet D Wu Z Marx CJ Vorholt JA Olson MV Kaul R Weissenbach J Medigue C Lidstrom ME Methylobacterium genome sequences: a reference blueprint to investigate microbial metabolism of C1 compounds from natural and industrial sources PLoS One 2009 3 5 e5584 10.1371/journal.pone.0005584 19440302 PMC2680597

