# Supplementary material for: Mercurial-resistance determinants in Pseudomonas strain K-62 plasmid pMR68
Source: AMB Express. 2013 Jul 28;3:41. doi: 10.1186/2191-0855-3-41 (PMC3737084; doi:10.1186/2191-0855-3-41)
Supplement: Additional file 1: Table S1 — Oligonucleotide primers used in this study. [file 2191-0855-3-41-S1.pdf]

Additional file 1: Table S1 Oligonucleotide primers used in this study

Primer	Oligonucleotide (5'---> 3')
1U-68kb-15850	TGACGAAGAAGCACTGACGG
2U-68kb-18520	CCGGAGTTGTGTCAATCCATC
3U-68kb-19421	GTGGTCGTGTCCATCTGCTC
4U-68kb-38451	GCCACCAACACGATGATCC
5U-68kb-42220	ACCGACAAACTCGCAGC
6U-68kb-67929	CGTAGTGCCCGAATTGCTC
7U-68kb-70484	CGAGTGAACCAGCCAAAGC
8L-68kb-16600	GCAGAATGGGATTGCGACC
9L-68kb-19216	CACCTTCGGCATCAAGCGTAG
10L-68kb-20259	GGGTGATGTAGGCCGTCTTG
11L-68kb-39477	CGATTTGACGAACGCGAGC
12L-68kb-43396	CGCTGACCTCGAAACAGAC
13L-68kb-68480	ATCGACCCATTCGAGGGTG
14L-68kb-457	GTAGCCCTGATCGACGAAG
16U-68kb-2393	GCTTGTTGCCGTCATCGCTTTCTGG
21L-68kb-9566	GGTAGTAAAGAGTGCCGCCGGAGTG
