# Supplementary material for: Mercurial-resistance determinants in Pseudomonas strain K-62 plasmid pMR68
Source: AMB Express. 2013 Jul 28;3:41. doi: 10.1186/2191-0855-3-41 (PMC3737084; doi:10.1186/2191-0855-3-41)
Supplement: Additional file 2: Table S2 — Summary of location of predicted coding regions on plasmid pMR68. [file 2191-0855-3-41-S2.pdf]

Additional file 2: Table S2 Summary of location of predicted coding regions on plasmid pMR68

Orf No.	Gene	pMR68 co-coordinates (5'-3')	Length (bp)	No. of amino acids	Sequence identity to best homolog	MexAM1_META locus tag
Orf 1	*doxX*	1316-1789	474	158	66% to DoxX from *Methylobacterium radiotolerans* JCM 2831	
Orf 2		2493-3539	1047	349	43% to YVTN family beta-propeller repeat from *Candidatus Solibacter usitatus* Ellin6076	
Orf 3	*merR*	4464-3967	498	166	73% to ZntR from *Methylobacterium extorquens* AM1	MexAM1_META1p2631
Orf 4		4322-5545	1224	408	H.P.	MexAM1_META1p2632
Orf 5		5659-6078	420	140	63% to Phosphoribosyl-AMP cyclohydrolase from *Parvibaculum lavamentivorans* DS-1	
Orf 6	*merT1*	6176-6577	402	134	76% to MerT from *Methylobacterium extorquens* AM1	MexAM1_META1p2633
Orf 7	*merP1*	6609-6944	336	112	76% to MerP from *Methylobacterium extorquens* AM1	MexAM1_META1p2634
Orf 8	*merF*	6966-7226	261	87	47% to MerF from *Gluconacetobacter xylinus* NBRC 3288	
Orf 9	*merA*	7226-8662	1437	479	84% to MerA from *Methylobacterium extorquens* AM1	MexAM1_META1p2635
Orf 10	*merB1*	8702-9472	771	257	40% to MerB from *Hyphomicrobium denitrificans* 1NES1	
Orf 11		10189-10770	582	194	81% to Transposase of ISMdi26, IS5 family from *Methylobacterium extorquens* DM4	
Orf 12		10913-11446	534	178	41% to Phosphopantothenoylcysteine decarboxylase from *Pedosphaera parvula* Ellin514	
Orf 13		11528-12802	1275	425	H.P.	
Orf 14		12977-13285	309	103	91% to Transposase IS3/IS911 family protein, partial from *Methylobacterium extorquens* DSM 13060	
Orf 15		13285-14016	732	244	89% to putative transposase, partial from *Methylobacterium extorquens* DSM 13060	
Orf 16		13514-14203	690	230	93% to Integrase catalytic region from *Methylobacterium extorquens* DSM 13060	
Orf 17		14067-14381	315	105	82% to Transposase IS3/IS911 family protein from *Methylobacterium chloromethanicum* CM4	
Orf 18		15814-14606	1209	403	97% to Transposase IS116/IS110/IS902 family protein from *Methylobacterium extorquens* DSM 13060	
Orf 19		15539-16111	573	191	H.P.	
Orf 20		18960-16144	2817	939	65% to Transposase Tn3 family protein from *Acidiphilium cryptum* JF-5	
Orf 21		16276-18840	2565	855	H.P.	
Orf 22		19725-19135	591	197	83% to resolvase domain-containing protein from *Acidiphilium cryptum* JF-5	
Orf 23		19950-20534	585	195	91% to Integrase catalytic region from *Methylobacterium extorquens* DSM 13060	
Orf 24		21162-20746	417	139	90% to Transposase and inactivated derivatives from *Magnetospirillum magnetotacticum* MS-1	
Orf 25		21161-23563	2403	801	62% to site-specific recombinase/DNA invertase from *Aromatoleum aromaticum* EbN1	
Orf 26		23446-22583	864	288	85% to Transposase IS4 family protein from *Methylobacterium extorquens* PA1	
Orf 27		24406-25458	1053	351	75% to Integrase catalytic region from *Methylobacterium nodulans* ORS 2060	
Orf 28		25421-27070	1650	550	H.P.	
Orf 29		28328-28942	615	205	66% to resolvase-like protein from *Agrobacterium tumefaciens* F2	
Orf 30		29408-28989	420	140	H.P.	
Orf 31		29650-29411	240	80	60% to prevent-host-death family protein from *Mesorhizobium sp.* BNC1	
Orf 32		30474-29989	486	162	90% to transposase from *Methylobacterium extorquens* DSM 13060	
Orf 33		30947-30567	381	127	83% to transposase from *Methylobacterium extorquens* DSM 13060	
Orf 34		31836-33500	1665	555	H.P.	MexAM1_META1p1874
Orf 35		34017-33520	498	166	H.P.	MexAM1_META1p1873
Orf 36		34864-34124	741	247	H.P.	MexAM1_META1p1872
Orf 37		35378-34872	507	169	H.P.	MexAM1_META1p1871
Orf 38		35584-35883	300	100	H.P.	
Orf 39		36192-38822	2631	877	76% to conjugative relaxase domain-containing protein from *Methylobacterium radiotolerans* JCM 2831	
Orf 40		39485-38892	594	198	H.P.	
Orf 41		40422-39640	783	261	H.P.	
Orf 42		41112-41768	657	219	36% to ParA/MinD from *Acetobacter pasteurianus subsp. pasteurianus* LMG 1262	
Orf 43		42446-42676	231	77	86% to CopG/Arc/MetJ family protein from *Agrobacterium tumefaciens* 5A	
Orf 44		42648-43187	540	180	86% to Transposase and inactivated derivatives from *Magnetospirillum magnetotacticum* MS-1	
Orf 45		43464-43823	360	120	61% to Transposase and inactivated derivatives from *Magnetospirillum magnetotacticum* MS-1	
Orf 46		45118-43508	1611	537	73% to Transposase IS66 from *Xanthobacter autotrophicus* Py2	
Orf 47		45516-45172	345	115	86% to IS66 Orf2 family protein from *Rhodomicrobium vannielii* ATCC 17100	
Orf 48		45926-45516	411	137	70% to Transposase and inactivated derivatives from *Magnetospirillum magnetotacticum* MS-1	
Orf 49		46885-46385	501	167	H.P.	
Orf 50		48114-47302	813	271	64% to glucose 1-dehydrogenase from *Candidatus Chloracidobacterium thermophilum* B	
Orf 51		49253-48138	1116	372	53% to FAD-dependent pyridine nucleotide-disulfide oxidoreductase from *Afipia sp.* 1NLS2	
Orf 52		50920-49565	1356	452	H.P.	MexAM1_META1p2630
Orf 53		53051-50970	2082	694	45% to glycoside hydrolase 15-related from *Methylobacterium radiotolerans* JCM 2831	
Orf 54		53946-53245	702	234	H.P.	
Orf 55		54717-54109	609	203	H.P.	
Orf 56		56455-55538	918	306	89% to Transposase of ISMdi25, IS30 family from *Methylobacterium extorquens* DM4	
Orf 57		55910-56473	564	188	58% to Integrase, catalytic region from *alpha proteobacterium* BAL199	
Orf 58		56730-56215	516	172	H.P.	
Orf 59		57264-57830	567	189	89% to Integrase catalytic region from *Methylobacterium sp.* 4-46	
Orf 60	*sigD*	57874-58428	555	185	62% to SigD gene product from *Bradyrhizobium japonicum* USDA 110	
Orf 61		58428-59066	639	213	H.P.	
Orf 62	*merT2*	59285-59686	402	134	67% to MerTfrom *Brevundimonas diminuta* ATCC 11568	
Orf 63	*merP2*	59725-60057	333	111	67% to MerP from *Methylobacterium extorquens* AM1	MexAM1_META1p2634
Orf 64		60403-61101	699	233	H.P.	
Orf 65		61542-61354	189	63	83% to putative insertion sequence transposase-like protein from *Methylobacterium populi* BJ001	
Orf 66		61858-62238	381	127	73% to Transposase from *Methylobacterium nodulans* ORS 2060	
Orf 67		62841-64118	1278	426	82% to Transposase IS66 from *Methylobacterium nodulans* ORS 2060	
Orf 68		65156-64134	1023	341	92% to Transposase IS116/IS110/IS902 family protein from *Methylobacterium extorquens* DSM 13060	
Orf 69		64683-65426	744	248	H.P.	
Orf 70	*rep*	65092-65607	516	172	85% to putative transposase of insertion sequence from *Methylobacterium nodulans* ORS 2060	
Orf 71		65775-65626	150	50	94% to putative insertion sequence transposase-like protein from *Methylobacterium populi* BJ001	
Orf 72	*merB2*	67205-66369	837	279	44% to MerB from *Streptomyces sp.* E14	
Orf 73		68058-68582	525	175	83% to Transposase from *Azospirillum sp.* B510	
Orf 74		69292-70512	1221	407	38% to putative adenylate cyclase protein from *Stappia aggregata* IAM 12614	
Orf 75		70923-700	798	266	79% to Transposase IS4 family protein from *Methylobacterium populi* BJ001	
